# P-1209. Changes in Antiviral Prescription for Children with Influenza in U.S. Emergency Departments During the COVID-19 Pandemic: New Vaccine Surveillance Network (NVSN), 2016-2023

**DOI:** 10.1093/ofid/ofae631.1391

**Published:** 2025-01-29

**Authors:** Tess Stopczynski, Olla Hamdan, Justin Z Amarin, James W Antoon, Laura S Stewart, James Chappell, Andrew J Spieker, Eileen J Klein, Janet A Englund, Geoffrey A Weinberg, Peter G Szilagyi, John V Williams, Marian G Michaels, Julie A Boom, Leila C Sahni, Mary A Staat, Elizabeth P Schlaudecker, Jennifer E Schuster, Rangaraj Selvarangan, Christopher J Harrison, Ayzsa Tannis, Heidi L Moline, Samantha M Olson, Natasha B Halasa

**Affiliations:** Vanderbilt University Medical Center, Nashville, Tennessee; Vanderbilt University Medical Center; Division of Pediatric Infectious Diseases, Nashville, Tennessee; Vanderbilt University Medical Center, Nashville, Tennessee; Vanderbilt University Medical Center, Nashville, Tennessee; Vanderbilt University Medical Center, Nashville, Tennessee; Vanderbilt University Medical Center, Nashville, Tennessee; Vanderbilt University Medical Center, Nashville, Tennessee; University of Washington School of Medicine, Seattle, Washington; Seattle Children’s Hospital, Seattle, Washington; University of Rochester School of Medicine & Dentistry, Rochester, NY; UCLA School of Medicine, Agoura Hills, California; University of Pittsburgh, Pittsburgh, Pennsylvania; UPMC Children's Hospital of Pittsburgh, Pittsburgh, Pennsylvania; Texas Children’s Hospital, Houston, Texas; Baylor College of Medicine and Texas Children’s Hospital, Houston, Texas; Cincinnati Children’s Hospital Medical Center, Cincinnati, Ohio; Cincinnati Children's Hospital Medical Center, Cincinnati, Ohio; Children’s Mercy Kansas City, Kansas City, Missouri; Children’s Mercy Kansas City, Kansas City, Missouri; Children's Mercy Hospital, Kansas City, Missouri; Centers for Disease Control and Prevention, Atlanta, Georgia; Centers for Disease Control and Prevention, Atlanta, Georgia; Centers for Disease Control and Prevention, Atlanta, Georgia; Vanderbilt University Medical Center, Nashville, Tennessee

## Abstract

**Background:**

Despite recommendations from CDC and ACIP, antiviral prescription for children who are at higher risk of severe influenza (including those < 5 years and those with certain underlying conditions) in emergency departments (EDs) remains suboptimal. This study assessed changes in antiviral prescription for children at higher risk of severe influenza in EDs from the pre-COVID-19-pandemic period to the COVID-19 pandemic period.

Demographic and clinical characteristics of children at higher risk of severe influenza(1) enrolled in the ED with laboratory confirmed influenza, stratified by pandemic periods (N=2,5001).

**Figure:**
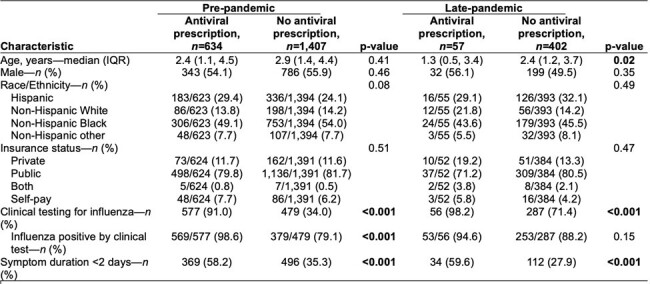

(1) Defined as children <5 years old and/or those with any underlying medical condition.

**Methods:**

Data from NVSN, a 7-site, prospective surveillance study of acute respiratory illnesses, were analyzed. Children at higher risk for severe influenza in EDs with confirmed influenza by research or clinical testing were included. We compared children who received antiviral prescriptions with those who did not. The study period was categorized into pre-pandemic (12/01/2016 to 03/31/2020) and late pandemic (07/01/2021 to 03/31/2023) periods. We used mixed-effects Poisson regression to compare antiviral prescription incidence proportions before the pandemic and during the late pandemic period. Mixed-effects logistic regression was used to evaluate factors associated with antiviral prescription during the late pandemic period among children at higher risk.

Incidence proportion ratios from mixed-effects Poisson model(1) among children at higher risk of severe influenza (N=2,500).

**Figure:**
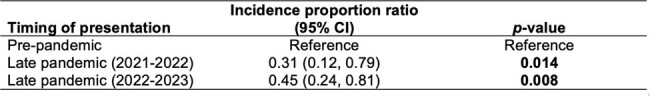

(1) The estimates presented are from a mixed-effects Poisson model with a log link and random intercepts for study sites, with outcome as number of children with an antiviral prescription.

**Results:**

A total of 3,436 children in the ED tested positive for influenza, of whom 2,500 (73%) were classified as higher risk for severe influenza. Pre-pandemic, 31% were prescribed antivirals compared to 12% during the late pandemic among those at higher risk (**Table 1**). Prescription of antivirals for children at higher risk decreased by 69% in 2021-2022 and by 55% in 2022-2023 compared to the pre-pandemic (**Table 2, Figure 1**). Symptom duration and clinical influenza testing were significantly associated with antiviral prescription among children at higher risk during the late pandemic period (**Figure 2**).

Percent of antiviral prescriptions among children at higher risk of severe influenza illness presenting to the emergency departments of seven children’s hospitals, stratified by clinical influenza testing (N=2,500).

**Figure:**
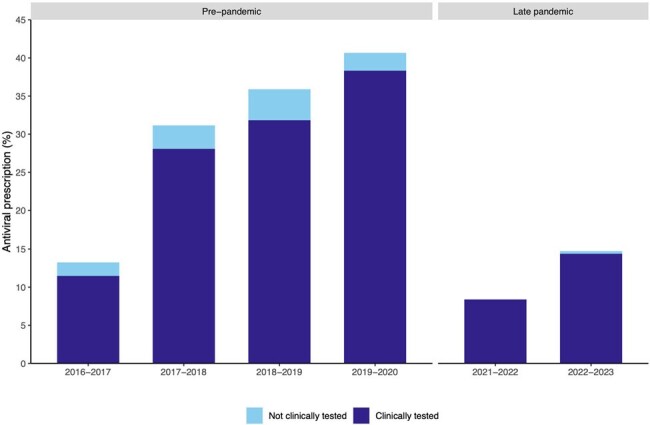

**Conclusion:**

Influenza antiviral prescription among children at higher risk for severe influenza in EDs decreased significantly during the COVID-19 pandemic compared to the pre-pandemic, despite treatment recommendations. While clinical testing has increased during the COVID-19 pandemic and is associated with prescription of influenza antivirals, prescription remains low. Efforts are needed to improve antiviral prescriptions for children at higher risk for severe influenza in EDs.

Adjusted odds ratios of antiviral prescription among children at higher risk of severe influenza illness presenting to the emergency departments of seven children’s hospitals during the pandemic period (2021-2023; N=459).

**Figure:**
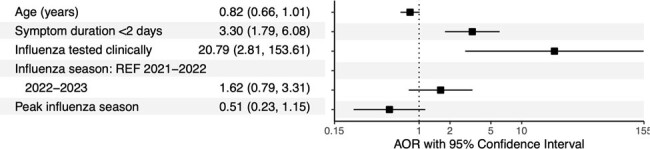

We used logistic regression to compare the odds of antiviral prescription, adjusting for age, symptom duration, clinical influenza testing (rapid antigen or PCR), influenza season, peak influenza season, and study site as a random effect.

**Disclosures:**

**James W. Antoon, MD, PhD, MPH**, AstraZeneca: Advisor/Consultant|NIH: Grant/Research Support **James Chappell, MD, PhD**, Merck: Grant/Research Support **Janet A. Englund, MD**, Abbvie: Advisor/Consultant|AstraZeneca: Advisor/Consultant|AstraZeneca: Grant/Research Support|GlaxoSmithKline: Advisor/Consultant|GlaxoSmithKline: Grant/Research Support|Meissa Vaccines: Advisor/Consultant|Merck: Advisor/Consultant|Pfizer: Board Member|Pfizer: Grant/Research Support|Pfizer: Speaker at meeting|SanofiPasteur: Advisor/Consultant|Shinogi: Advisor/Consultant **Geoffrey A. Weinberg, MD**, Inhalon: Advisor/Consultant|Merck & Company: Honoraria for textbook chapter preparation **Mary A. Staat, MD, MPH**, Cepheid: Grant/Research Support|Merck: Grant/Research Support|Pfizer: Grant/Research Support|Up-To-Date: Honoraria **Elizabeth P. Schlaudecker, MD, MPH**, Pfizer: Grant/Research Support|Sanofi Pasteur: Advisor/Consultant **Rangaraj Selvarangan, BVSc, PhD, D(ABMM), FIDSA, FAAM**, Abbott: Grant/Research Support|Abbott: Honoraria|BioMerieux: Grant/Research Support|Cepheid: Grant/Research Support|Diasorin: Grant/Research Support|GSK: Advisor/Consultant|Hologic: Grant/Research Support|Luminex: Grant/Research Support|Qiagen: Grant/Research Support **Christopher J. Harrison, MD**, GSK: Grant/Research Support|Medscape: Honoraria|Merck: Grant/Research Support|Pfizer: Grant/Research Support|UpToDate: Honoraria **Natasha B. Halasa, MD, MPH**, Merck: Grant/Research Support

